# Diagnostic Accuracy of Lever Sign Test in Acute, Chronic, and Postreconstructive ACL Injuries

**DOI:** 10.1155/2019/3639693

**Published:** 2019-06-09

**Authors:** Tahsin Gürpınar, Barış Polat, Ayşe Esin Polat, Engin Çarkçı, Yusuf Öztürkmen

**Affiliations:** ^1^Istanbul Training and Research Hospital, Department of Orthopedics and Traumatology, Istanbul, Turkey; ^2^University of Kyrenia, Faculty of Medicine, Department of Orthopaedics and Traumatology, Kyrenia, Cyprus; ^3^Dr. Akçiçek State Hospital, Department of Orthopaedics and Traumatology, Kyrenia, Cyprus

## Abstract

**Background:**

The aim of this study is to determine the diagnostic accuracy of lever sign test in acute, chronic, and postreconstructive ACL injuries.

**Methods:**

In total, 78 patients (69 male, 9 female) were subjected to clinical instability tests including Lachman, anterior drawer, pivot shift, and lever sign when an injury of the ACL was suspected. All tests were performed bilaterally in all patients in acute, chronic period and patients who underwent surgery after the anaesthesia and after the reconstruction at the last follow-up by two senior orthopaedic surgeons. MRI was taken from all patients and MRI image was taken as the reference test when evaluating the accuracy of the tests.

**Results:**

The mean age of patients was 26.2±6.4 years (range, 17-44 years). Sensitivity and accuracy values of the Lachman, anterior drawer, pivot shift, and lever tests in the acute phase were calculated as 80.6%, 77.4%, 51.6%, 91.9% and 76.9%, 75.6%, 60.3%, 92.3%, respectively, and in the chronic (preanaesthesia) phase were calculated as 83.9%, 79.0%, 56.5%, 91.9% and 80.8%, 78.2%, 64.1%, 92.3%, respectively. Lachman, anterior drawer, pivot shift, and lever sign Acute's significant [AUC: 0.716, 0.731, 0.727, 0.928, respectively] activity were observed in the prediction of ACL rupture in MRI.

**Conclusion:**

An ideal test to diagnose the integrity of the ACL should be easy to perform and reproducible with high sensitivity and specificity. From this perspective, the lever test seems to be a good test for clinicians in acute, chronic and postreconstructive ACL injuries.

## 1. Introduction

The diagnosis of ACL rupture is generally made by anamnestic findings, physical examination tests, MRI imaging, and arthroscopy. Many physical examination tests have been proposed to assess ACL stability and the most commonly used are the Lachman test, anterior drawer test, and pivot shift test [1, 2]. The sensitivity, specificity, and shortcomings of these tests have been widely studied and they are commonly used for both diagnosis and follow-up after the surgery [3–5]. However, it is known that they can be influenced by many factors [6]. Acute injuries usually lead to reactive synovitis, hemarthrosis, and knee swelling, which may cause the patient to be guarded during the examination due to fear of pain or subluxation. In addition, partial ruptures may be harder to diagnose than complete ruptures due to the stability provided by the remaining fibres and the presence of meniscal tear may affect the physical examination tests results [4, 7]. Lever sign is a new test introduced by Dr. Lelli to overcome these shortcomings [8]. However, there are only limited studies investigating the sensitivity and specificity of lever sign and the results are dissimilar among different studies [8–12]. In addition, the value of the lever sign test for postoperative follow-up was not to be reported yet. The aim of this study is to determine the diagnostic accuracy of lever sign test in acute, chronic and postreconstructive ACL injuries.

## 2. Methods

All consecutive patients referred to the Istanbul Training and Research Hospital after sustaining an acute knee injury were subjected to clinical instability tests including Lachman, anterior drawer, pivot shift and lever sign when an injury of the ACL was suspected between January 2016 and January 2018. All tests were independently performed bilaterally and by two senior orthopaedic specialists. The patients were reexamined until a consensus occurred in the case of a disagreement between the examiners. An audible pop at the time of injury, immediate swelling or hemarthrosis, feeling of instability and subluxation episodes are considered to be possible signs of ACL injury. The examination results were recorded and patients were followed up to evaluate the results of MRI and any later possible surgical procedures.

Only patients who had contact or noncontact knee injuries up to 2 weeks prior to the examination and who did not have any previous history of knee injury were included in this study. A total of 15 patients were excluded in the study. There were five patients with multiple ligamentous injury, four patients with severe arthritic changes (joint space narrowing, grade 3 or greater cartilage injury on MRI), two patients who underwent prior ACL reconstruction on the contralateral side, one patient sustained contralateral knee injury at the same time, and three patients did not present for follow-up. Finally, a total of 78 patients met the inclusion criteria. All patients who met the inclusion criteria were asked to complete a written consent form before participating in the study. This study and the associated informed consent procedures were approved by the medical ethical review committee of Istanbul Training and Research Hospital (Reference No: 1522). When hemarthrosis was present, it was drained before the examination to avoid external factors. MRI was taken for all patients because of their anamnestic findings and MRI was used as our reference test. The surgical decision was based on combination of clinical and MRI findings. Arthroscopic findings including meniscal tears, partial or total rupture of the ACL were also noted. All clinical examinations were repeated after anaesthesia prior to the surgery (chronic) and after reconstruction at the last follow-up if patients underwent arthroscopic reconstruction.

All tests were performed on the contralateral noninjured leg of the patients. Even of the examination test was bilateral positive, if the affected knee anterior cruciate ligament was intact in knee MRI, the test was considered as false positive. The contralateral knees did not have a history of trauma and therefore ACL was assumed to be intact in these knees. All tests were assessed based on this assumption and it was not confirmed with MRI or arthroscopic surgery.

In this study, the efficacy of four physical examination tests was evaluated in both the acute and preanaesthesia (chronic) period, as well as postanaesthesia and postoperative at the last follow-up period in 49 patients who underwent surgery. Preanaesthesia (chronic) period examination was performed 4 weeks after injury in both operated and nonoperated group. Subanalyses were performed based on the presence of meniscus tear according to MRI.

### 2.1. MRI Evaluation

MRI was taken from all patients because of the amnestic findings of instability. All examinations were performed on a 1.5 T whole body MRI system (Signa HDxt, GE Medical Systems, Milwaukee, Wisconsin, USA) with a 33 mT/m maximum gradient capacity. Images were evaluated by an experienced musculoskeletal radiologist and an orthopaedic surgeon. MRI showing an ACL tear was used as the gold standard for diagnosis of an ACL tear.

### 2.2. Instability Tests

**Figure 1:**
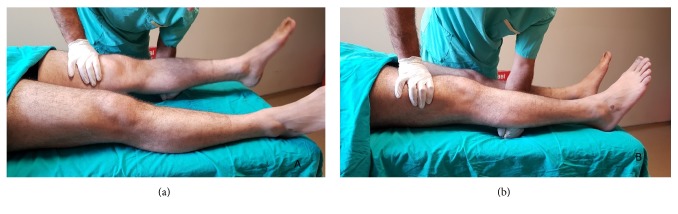
The view of the intact ACL (a) and ACL injury (b) side.

The lever sign test was applied as described by Lelli et al. [8]. The patient was in a supine position on a hard examination table with both legs extended and the clinician placed his fist below the proximal third of the cruris as a fulcrum. This manoeuvre brings the knee slightly into flexion. With the other hand, the clinician applied a force over the distal third of the quadriceps downwards onto the thigh. In an intact knee, ACL completes the lever arm and the downward force on the quadriceps therefore, creates a rotational movement on the knee joint and the heel rises up off the examination table. With a ruptured ACL, the ability to offset the force of gravity on the lower leg is compromised and the tibial plateau slides anteriorly with respect to the femur. If the heel does not rise up off the examination table and the tibial plateau slides anteriorly instead, the test is considered positive (Figure 1).

Lachman, anterior drawer, and pivot shift tests were routinely performed tests as described in the literature [13, 14].

### 2.3. Surgical Technique

After the decision to perform arthroscopic reconstruction was made, all patients were required to wait until the knee was ready for surgery. The criteria for having the injured knee ready for surgery were determined as follows: full range of motion, no swelling or effusion of the knee, full quadriceps strength, and ability to walk without pain or limping.

All surgeries were performed by the first author in a single institution. After diagnostic arthroscopy for confirming the ACL injury, hamstring autografts (both semitendinosus and gracilis) were harvested, prepared, and double looped. The anatomic footprints were prepared with an arthroscopic shaver and radiofrequency device. The femoral tunnel was opened freehand through the accessory medial portal and the tibial tunnel was opened by using a Smith & Nephew ACUFEX Director Drill Guide System. The graft was passed and fixed with an Endobutton on the femoral side and with a Smith & Nephew interference screw and U staple on the tibial side. If meniscal tears were present, meniscal repair or partial meniscectomies were performed.

### 2.4. Statistics

In the descriptive statistics of the data, mean, standard deviation, median lowest, highest, frequency, and ratio values were used. The distribution of the variables was measured with the Kolmogorov-Smirnov test. The chi-square test was used for the analysis of qualitative independent data and the Fischer test was used when the chi-square test conditions were not met. Effect level and cut-off value were investigated with ROC curve. The SPSS 22.0 program was used in the analysis. The alpha level of significance was accepted as p <0.05.

Sensitivity, specificity, positive predictive value (PPV), negative predictive value (NPV), and accuracy value were calculated as follows: Sensitivity = true positives/(true positives + false negatives); Specificity = true negatives/(true negatives + false positives); PPV = true positives/(true positives + false positives); NPV = true negatives/(true negatives + false negatives); Accuracy = (true positives + true negatives)/(true positives + false positives + true negatives + false negatives).

Interobserver agreement was assessed using the kappa coefficient (*κ*) statistical test. The *κ*-value between 0.8 and 1 was considered as perfect agreement.

## 3. Results

In total, 78 patients were suspected to have an ACL injury who had no previous history of any knee injury. In terms of patient information, 9 of the patients were female (11.5 %) and 69 were male (88.5 %), with a mean age of 26.2±6.4 years (range, 17-44 years) at the initial clinic visit. There were 36 left knees (46.2 %) and 42 right knees (53.8 %). The trauma mechanism of 68 (87.1%) of the cases was sports injury. Other causes included: 4 motor vehicle injuries, 2 patients fell down stairs, 2 fell from a height, 1 knee distortion on a fight, and 1 was caused by knee distortion while walking on the street. The mean injury time to acute examination time was 5.9±4.0 (range, 0-14) days. All patients had acute traumatic knee injury and 19 (24.4 %) had severe hemarthrosis, which was drained before examination. Furthermore, 16 patients' ACL were intact according to MRI, 49 of the patients underwent arthroscopic ACL reconstruction, while the remaining 13 patients refused surgery or they had not been operated on at the time of the study (Table 1). Physical examination tests was repeated in 49 operated patients with a mean follow-up of 21,2 months (range, 12-32).

### 3.1. MRI Findings

In total, 78 patients were suspected to have an ACL injury and underwent MRI. Total or partial ACL injury was confirmed in 62 of them on MRI. 50 of the ruptures were considered as total, whereas the remaining 12 were considered as partial tears. No distinction between anteromedial (AM) or posterolateral (PL) bundle tear was made on MRI for partial tears. 15 of the patients had lateral and 11 patients had medial meniscus tears (Table 1).

### 3.2. Surgical Findings

In the surgical group, MR was compared with direct arthroscopic visualization and when the 49 patients who underwent arthroscopy were analysed, positive anamnestic finding with MRI had a 100% sensitivity, 100% specificity, and 100% PPV. 49 patients underwent arthroscopic reconstruction, where total tear was determined in 43 cases and partial tear in 6 cases (2 AM bundle and 4 PL bundle). Six patients who were considered to have partial tears on MRI were found to have total ruptures on arthroscopy and for this reason, we did not evaluate the total rupture subgroups with partial rupture of MRI in terms of physical examination tests. In total 8 lateral and 7 medial meniscus tears were sutured. From the remaining patients, 7 had lateral and 4 had medial meniscus tears that were not suitable for repair; consequently, partial meniscectomy was performed on those patients.

### 3.3. Diagnostic Tests Results

A significant correlation was found between ACL injury in MRI and acute, chronic periods of all four physical examination tests. The highest measure of agreement kappa, area under curve (AUC), sensitivity, PPV, NPV, and accuracy were obtained from the assessments with the lever test. In the acute and chronic periods, the measure of agreement kappa of the results of the evaluation of the lever test was 0.784. The sensitivity and accuracy values of the Lachman, anterior drawer, pivot shift, and lever tests in the acute phase were calculated as 80.6%, 77.4%, 51.6%, 91.9% and 76.9%, 75.6%, 60.3%, 92.3%, respectively. The sensitivity and accuracy values of the Lachman, anterior drawer, pivot shift, and lever tests in the chronic (preanaesthesia) phase were calculated as 83.9%, 79.0%, 56.5%, 91.9% and 80.8%, 78.2%, 64.1%, 92.3%, respectively (Table 2).

**Figure 2:**
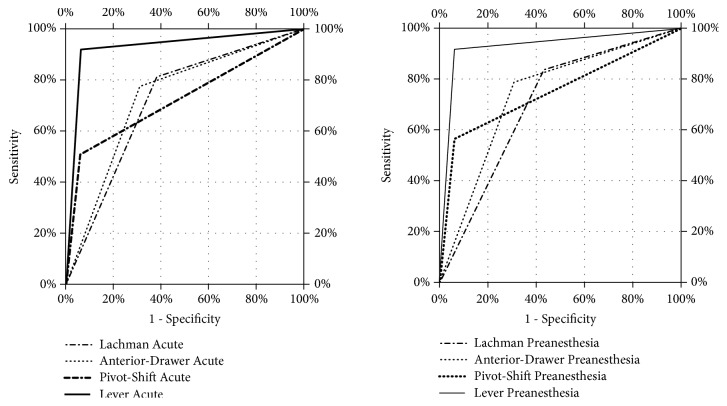
A graphical presentation of the relationship between both sensitivity and specificity of 4 examination tests in acute and chronic (preanaesthesia) period.

Lachman, anterior drawer, pivot shift, and lever sign Acute's significant [AUC: 0.716, 0.731, 0.727, 0.928, respectively] activity was observed in the prediction of ACL rupture in MRI. Lachman, anterior drawer, pivot shift, and lever sign Chronic's (preanaesthesia) significant [AUC: 0.701, 0.739, 0.751, 0.928, respectively] activity was observed in the prediction of ACL rupture in MRI (Table 3 and Figure 2).

In the acute period, the measure of agreement kappa, sensitivity, PPV, specificity, NPV and accuracy ratios of the lever test in patients without meniscal tears were higher than the meniscus rupture group (Table 4).

Sensitivities of Lachman, anterior drawer, pivot shift, and lever tests were calculated as 89.7%, 79.5%, 77.5%, 91.9%, respectively, in the postanaesthesia period of patients who were operated on for ACL rupture. The specificities of Lachman, anterior drawer, pivot shift, and lever tests were calculated as 96%, 91.9%, 96%, 96%, respectively in the postoperative last follow-up period of the patients who were operated on for ACL rupture (Table 5).

Interobserver agreement (*κ*) was 0.89 for the lever sign test, 0.86 for the Lachman test, 0.81 for the pivot shift test, and 0.83 for the anterior drawer test.

## 4. Discussion

This study investigated the diagnostic properties of the lever sign test regarding ACL ruptures. The results indicate that the lever sign test is highly sensitive and specific to ACL injury and can easily be performed in both acute and chronic periods after the injury with high interobserver reliability. Most importantly, we found that this new test is more sensitive in acute cases than the Lachman test, which is commonly considered as the most sensitive test for ACL injury [5, 15]. With respect to the Lachman, anterior drawer (AD), and pivot shift tests, our study showed comparable results to previous meta-analyses. A recent meta-analysis with pooled results from 16 studies showed that the overall sensitivity of the anterior drawer test was 0.725, and the specificity was 0.927 [15]. For the Lachman test, the overall sensitivity was 0.871 and the specificity was 0.97. For the pivot shift test, the overall specificity was 0.975; however, the sensitivity was 0.490. These are comparable to our results for the Lachman test, AD and pivot shift test.

The lever sign test was introduced by Lelli at al. to overcome the lowered sensitivity of previously described tests, particularly for acute ACL injuries [8]. Acute ACL injuries are generally regarded as more difficult to diagnose due to pain, reactive synovitis, haemarthrosis, and swelling [16, 17]. In the literature, It has been reported that the sensitivity for acute injuries is 0.78 for the Lachman test, 0.22 for the anterior drawer test, and 0.89 for the pivot shift test [4]. However, in his own study, Lelli claimed that the lever sign test has 100% sensitivity for acute cases [8]. Nevertheless, only two studies after Lelli have investigated the sensitivity of the lever sign test in acute cases. Massey et al. [12] found the sensitivity of the lever sign test in acute cases to be 90% and the specificity to be 77%. However, Jarbo et al. [10] found a sensitivity of 63% and a specificity of 90%. In our study, we followed the same particular patient group in acute, chronic, and postoperative periods and we found the sensitivity of lever sign to be 91.9% and the specificity to be 93.8% in the acute period. This result was higher than the Lachman test, which was 80.6% in our study. In addition, we did not observe any difference in the sensitivity between the acute and chronic periods for the lever sign test (91.9% acute, 91.9% chronic). In contrast, all other tests had lowered sensitivity in the acute period. Among these tests, the sensitivity of the Lachman (80.6% acute, 83.9% chronic), anterior drawer (77.4% acute, 79.0% chronic), and pivot shift (51.6% acute, 56.5% chronic) test increased in the chronic period compared to the acute period. Therefore, our study shows that external factors such as pain, patient resistance, haemarthrosis, swelling, or time from the injury have minimal or no effect on the sensitivity of the lever sign test, but alter the sensitivity of the anterior drawer test the most. In addition, in the chronic period before and after anaesthesia, the sensitivity of the lever sign test was slightly higher than the Lachman test. We recommend that the lever sign test be performed, particularly in acute cases when an ACL injury is suspected.

Regardless of the time after injury, patient resistance is another factor that can alter the sensitivity of the tests. It is known that the sensitivity of the pivot shift test and anterior drawer test can be very different before and after anaesthesia [18]. In a meta-analysis, it has been reported that the sensitivity of the anterior drawer test and the pivot shift test increased from 38 to 63 % and 28 to 73 %, respectively after anaesthesia [19]. In this study, the sensitivity of the Lachman, anterior drawer test and the pivot shift test increased from 83.9% to 89.7%, from 79.0% to 79.5% and from 56.5% to 77.5%, respectively, after anaesthesia. However, did not observe any difference in the sensitivity of the lever sign test before and after anaesthesia (91.9% to 91.9%) similar to the results found by Deveci et al. [9]. Therefore, we conclude that the lever sign test can easily be performed regardless of patient resistance. This is important to distinguish the lever sign test among other tests, because the sensitivity of all other tests improved after anaesthesia.

The lever sign test is a new test and the ability to learn how to perform the test has not been evaluated. All authors mentioned that the test is easy to perform and has a higher ICC, but they found different results in a similar patient population [9–11]. Before starting this study, we assessed our ability on patients with proven ACL ruptures. One of the technical points we found important while learning how to perform the test is the rigidity of the examination table. The examination tables are not all standardized and some do not support the fist in a similar manner as the fulcrum on the lever sign test. When a posterior force is applied to the femur on a softer examination table, the fist can be embedded and it therefore does not work as a fulcrum and the heel does not rise. This can cause false negativity of the test and is an important factor to consider. To avoid this, Massey et al. [12] used a flat hard surface under the leg before the examination. Besides this, we found the test easy to perform it does not make the patient feel uncomfortable.

Another factor that can affect the accuracy of ACL tests is the presence of a meniscus tear. In a biomechanical study, Spang et al. [20] found a significant increase in tibial displacement relative to the femur after medial meniscectomy. It is also shown that menisci are secondary restraints of tibial anterior translation and one can therefore conclude that a meniscal tear can increase the instability of the knee. Speziali et al. [21] found that the testing accuracy decreased when both an ACL and meniscus tear were present. Furthermore, Massey et al. [12] found that the accuracy of the lever sign decreased from 89% to 74% in the presence of meniscal tears, which was statistically significant. In the present study the accuracy of the lever sign decreased from 96.2% to 84.6% when a meniscus tear was present in accordance with the literature. This could be due to differences between studies in terms of tear sizes and locations, presence of hematoma, and effusion or patient guarding. Different from Massey et al., we drained the hematoma or effusion if swelling was present before starting examination, which can impact the accuracy of the diagnostic tests. Similarly, Wang et al. [6] showed that joint aspiration can raise the sensitivity of physical examination for diagnosing acute ACL injury. The effect of meniscus tears on the accuracy of the lever sign test is not mentioned in other studies that have investigated the lever sign test.

We followed up the patients at least 6 months postoperatively and throughout this period, we evaluated the functional results and stability of the knees. We repeated the lever test in this period and the results of the lever sign test were correlated with functional outcomes. We did not send patients to MRI unless we found any instability; therefore, we could not compare the results of lever sign with MRI results. However, we compared the lever test specificity with the Lachman test specificity, which is commonly used after ACL reconstructions to assess the stability and we found similar results (96% and 96%) between these two tests [22–24]. Therefore, we believe that the lever sign test can also be used to evaluate the results after surgery. To our knowledge, it has not been used before to evaluate the clinical results.

There are some limitations to this study. For example, the number of females in the study was low and differences in terms of gender were not evaluated. The specificity of all tests were assessed according to the results of the contralateral noninjured leg however, contralateral knees were not evaluated with MRI or arthroscopic surgery. The testers were also not blinded to the patient history and therefore they looked for instability, but did not know the results of MRI. Additionally, we did not use KT-1000 or another contemporary technique to objectively quantify laxity because we can not obtain this kind of machine. On the other hand, one of the strongest points of this study is that we followed up the same patient group in acute and chronic stages; therefore, we could see the changes in the same patients in the chronic stage not in another patient group.

## 5. Conclusions

An ideal test for diagnosing the integrity of the ACL should be easy to perform and reproducible with high sensitivity and specificity. From this perspective, the lever test seems to be a good test for clinicians in acute, chronic, and postreconstructive ACL injuries.

## Figures and Tables

**Table 1:** Patients demographics and the condition of the ACL, meniscus structures of patients on MRI.

		Min-Max			Median	Mean±sd/n-%		
Age		17 - 44			25.00	26.17 ± 6.445		

Sex	Female					9		11.5%
	Male					69		88.5%

Injury Time to Examination		0 - 14			5.00	5.88 ± 4.026		

Side	Left					36		46.2%
	Right					42		53.8%

MRI-ACL	Intact					16		20.5%
	P.Rupture					12		15.4%
	Rupture					50		64.1%

MRI-Meniscus Tear	(-)					52		66.7%
	(+)					26		33.3%

Surgery ACL	Not Operated					29		37.2%
	Reconstruction					49		62.8%

Hemarthrosis	(-)					59		75.6%
	(+)					19		24.4%

**Table 2:** The effectiveness of 4 physical examination tests in acute and chronic (preanaesthesia) period.

		MR Rupture		Sensitivity	PPV	Specificity	NPV	Accuracy	Kappa	p
		(-)	(+)							
Lachman Acute	(-)	10	12	80.6%	89.3%	62.5%	45.5%	76.9%	0.379	0.001 ^K^
	(+)	6	50							

Anterior-Drawer Acute	(-)	11	14	77.4%	90.6%	68.8%	44.0%	75.6%	0.382	0.001 ^K^
	(+)	5	48							

Pivot-Shift Acute	(-)	15	30	51.6%	97.0%	93.8%	33.3%	60.3%	0.271	0.001 ^K^
	(+)	1	32							

Lever Acute	(-)	15	5	91.9%	98.3%	93.8%	75.0%	92.3%	0.784	<0.001 ^K^
	(+)	1	57							

Lachman Preanesthesia	(-)	11	10	83.9%	91.2%	68.8%	52.4%	80.8%	0.472	<0.001 ^K^
	(+)	5	52							

Anterior-Drawer Preanesthesia	(-)	12	13	79.0%	92.5%	75.0%	48.0%	78.2%	0.447	<0.001 ^K^
	(+)	4	49							

Pivot-Shift Preanesthesia	(-)	15	27	56.5%	97.2%	93.8%	35.7%	64.1%	0.313	0.001 ^K^
	(+)	1	35							

Lever Preanesthesia	(-)	15	5	91.9%	98.3%	93.8%	75.0%	92.3%	0.784	<0.001 ^K^
	(+)	1	57							

^K^ Kappa Test

PPV: Positive predictive value, NPV: Negative predictive value, MR: Magnetic resonance

**Table 3:** The effectiveness of 4 physical examination tests in acute and chronic (preanaesthesia) period.

	AUC	% 95 CI	p
Lachman Acute	0.716	0.564 - 0.868	0.008
Anterior-Drawer Acute	0.731	0.585 - 0.877	0.005
Pivot-Shift Acute	0.727	0.606 - 0.848	0.005
Lever Acute	0.928	0.849 - 1.000	<0.001
Lachman preanaesthesia	0.701	0.544 - 0.858	0.014
Anterior-Drawer preanaesthesia	0.739	0.594 - 0.884	0.003
Pivot-Shift preanaesthesia	0.751	0.635 - 0.867	0.002
Lever preanaesthesia	0.928	0.849 - 1.000	<0.001

ROC Curve

**Table 4:** Effect of the meniscus tear on the results of the lever test in acute period.

		MR Rupture		Sensitivity	PPV	Specificity	NPV	Accuracy	Kappa	p
		(-)	(+)							
*MRI-mensicus tear Yes*										
Lever Acute	(-)	5	3	85.0%	94.4%	83.3%	62.5%	84.6%	0.612	0.001 ^K^
	(+)	1	17							

*MRI-mensicus tear No*										
Lever Acute	(-)	10	2	95.2%	100%	100%	83.3%	96.2%	0.885	<0.001 ^K^
	(+)	0	40							

^K^ Kappa Test

**Table 5:** The sensitivity of 4 physical examination tests in postanaesthesia period and the specificity of 4 physical examination tests in postoperative last follow-up period.

		N (49)	sensitivity (%)
Postanaesthesia-Positive	Lachman	44	89.7
	Anterior-drawer	39	79.5
	Pivot-shift	38	77.5
	Lever	45	91.9
		N (49)	specificity (%)
Postop-Negative	Lachman	47	96.0
	Anterior-drawer	45	91.9
	Pivot-shift	47	96.0
	Lever	47	96.0

## Data Availability

The data used to support the findings of this study are included within the article.
